# Virtual darkness for agitation in dementia: The DARK.DEM randomized controlled trial

**DOI:** 10.1192/j.eurpsy.2025.1724

**Published:** 2025-08-26

**Authors:** L. I. I. Berge, T. E. G. Henriksen, S. V. Skagen, K. Nedreskår, V. Casadei, A. M. E. Mork, M. Patrascu, S. E. Fæø, E. Flo-Groeneboom

**Affiliations:** 1 Department of Global Public Health and Primary Care, University of Bergen, Bergen; 2 NKS Olaviken Gerontopsychiatric Hospital, Askøy; 3 Helse Fonna, Valen Hospital Trust, Valen; 4 Department of Global Public Health and Primary Care, University of Begen; 5 Department of nursing, Vid Specialized University; 6 Department of clinical psychology, University of Bergen, Bergen, Norway

## Abstract

**Introduction:**

This poster will outline the rationale, objective, methods and preliminary results of the DARK.DEM trial.

**Introduction:**

Behavioral and psychological symptoms of dementia (BPSD) such as agitation, psychosis and depression are prevalent, often treatment resistant and associated with reduced cognition, level of functioning, quality of life and mortality. In dementia, circadian rhythms become less robust, which potentiates BPSD. As such, *chronotheraphy*, i.e., interventions targeting the circadian rhythm, is promising. Bright light therapy can improve sleep and depressive symptoms in residents with dementia in nursing homes (Hjetland GJ, BMC Geriatrics, 2021). Intrinsically photosensitive retinal ganglion cells (*ipRGC)* monitor the perception of day and night and are maximally sensitive to light with short wavelength. This discovery paved the way for virtual darkness therapy, that is, solely exposure to light deprived of blue wavelengths in the evening and night. Virtual darkness is effective in reducing manic symptoms in persons with bipolar disorders (Henriksen TEG, Bipolar Disorders, 2016), however the symptom relieving effect in people with dementia has not been explored.

**Objectives:**

Develop and evaluate virtual darkness therapy to enhance treatment of agitation and other BPSDs in specialized dementia care.

**Methods:**

The DARK.DEM RCT will include patients from September 2024 to December 2027 at NKS Olaviken Gerontopsychatric Hospital, Norway.

**Inclusion criteria:**

dementia related agitation (CMAI ≥45), all etiologies and stages of dementia, age ≥50. Exclusion criteria: use of beta-blockers or melatonin, clinically significant pain (MOBID-2≥3), total blindness. A total of 72 patients will be randomized to treatment as usual or 14 days with add on treatment with blue light depleted environment from 19-08, provided with circadian lightning in secluded units. Primary outcome is 14 day change in CMAI, secondary outcomes include change in NPI-12, CSDD, QoL, ADL, use of psychotropic drugs and restraints, length of hospital stay.

**Results:**

We will present preliminary results on number of participants included, baseline characteristics related to age, sex, type dementia, level of symptoms, intervention effects and feasibility.

**Conclusions:**

The DARK.DEM trial will provide real word evidence for clinicians and stakeholders to determine if virtual darkness theraphy should be implemented for treatment of agitation in people with dementia.

**Disclosure of Interest:**

None Declared

